# Long-term clinical course and outcomes in patients with lymphangioleiomyomatosis

**DOI:** 10.1186/s12931-022-02079-6

**Published:** 2022-06-18

**Authors:** Hee-Young Yoon, Ho Jeong Kim, Jin Woo Song

**Affiliations:** 1 Division of Internal Medicine, Seoul North Municipal Hospital, 38, Yangwonyeok-ro, Jungnang-gu, Seoul, 02062 Republic of Korea; 2 Department of Pulmonary and Critical Care Medicine, Asan Medical Center, University of Ulsan College of Medicine, 88, Olympic-Ro 43-Gil, Songpa-Gu, Seoul, 05505 Republic of Korea

**Keywords:** Lymphangioleiomyomatosis, Prognosis, Respiratory function test, Patient outcome assessment, Rare diseases

## Abstract

**Background:**

Lymphangioleiomyomatosis (LAM) is a rare multisystemic disorder with various clinical manifestations. Despite the recognition of several prognostic factors, the long-term clinical course and prognosis of patients with LAM in the era of sirolimus therapy are not established.

**Methods:**

The clinical data of 104 patients with LAM were retrospectively analyzed. Death or lung transplantation was defined as the primary outcome. Disease progression (DP) was defined as a 10% absolute decline in forced expiratory volume in one second (FEV_1_).

**Results:**

The mean age of all patients was 40.3 years. Over a median follow-up period of 7.1 years, of all patients, 6.7% died and 1.9% underwent lung transplantation, while of 92 patients with serial lung function data, 35.9% experienced DP. The 5-year and 10-year overall survival rates were 93.0% and 90.9%, respectively. The multivariable Cox analysis revealed that older age (hazard ratio [HR]: 1.136, *P* = 0.025), lower FEV_1_ (HR: 0.956, *P* = 0.026) or diffusing capacity for carbon monoxide (HR: 0.914, *P* = 0.003), and shorter distance during the 6-min walk test (HR: 0.993, *P* = 0.020) were independent prognostic factors for mortality. A propensity score-matched comparative analysis performed between patients who received sirolimus therapy and those who did not, found no differences in survival, DP, complications, and lung function decline rate.

**Conclusions:**

Over a follow-up period of approximately 7 years, one-tenth of all patients experienced death, while one-third experienced DP. Older age, lower lung function, and reduced exercise capacity were associated with a poor prognosis in patients with LAM.

**Supplementary Information:**

The online version contains supplementary material available at 10.1186/s12931-022-02079-6.

## Background

Lymphangioleiomyomatosis (LAM) is a rare multisystemic disorder which mainly affects women of childbearing age [1, 2]. The clinical course of LAM is heterogenous, with a reported 10-year survival rate of 80–90% [3–6]. A study including 401 patients with LAM showed that the 10-year transplant-free survival rate was 86% [3]. Another study conducted on 217 patients with LAM, also reported 5-year and 10-year transplant-free survival rates of 94% and 85%, respectively [5]. Moreover, the 5-year and 10-year survival rates of 173 Japanese patients with LAM were 91% and 76%, respectively [6]. Patients with LAM experience dyspnea and a progressive decline in lung function, both of which eventually result in the need for lung transplantation. Johnson et al. found that the median time from symptom onset to grade 3 dyspnea according to the Medical Research Council Dyspnea Scale, was 9.3 years in patients from the United Kingdom (UK) with LAM (n = 72) [4].

Several prognostic factors in patients with LAM have been reported in previous studies [3, 5–8]. In a previous study, lower FEV_1_ and diffusing lung capacity for carbon monoxide (DLCO) were found to be poor prognostic factors for transplantation-free survival in the age-adjusted Cox analysis [5]. Of a total of 46 Japanese patients with LAM, the non-survivors were found to have a lower forced expiratory volume in one second/forced vital capacity (FEV_1_/FVC) ratio and a higher total lung capacity (TLC) than the survivors [7]. Moreover, delayed symptom onset and the presence of angiomyolipoma (AML) were associated with better survival in patients with LAM, whereas home oxygen use was associated with poorer survival [3]. However, the long-term clinical course and prognostic factors of LAM in the era of sirolimus therapy which has been recently introduced to the standard treatment, are not well defined. Moreover, the effect of sirolimus on the survival of patients with LAM in a real-world setting has not been investigated. This study aimed to evaluate the long-term clinical course, prognostic factors, and impact of sirolimus on the prognosis of patients with LAM.

## Study design and methods

### Study population

**Fig. 1:**
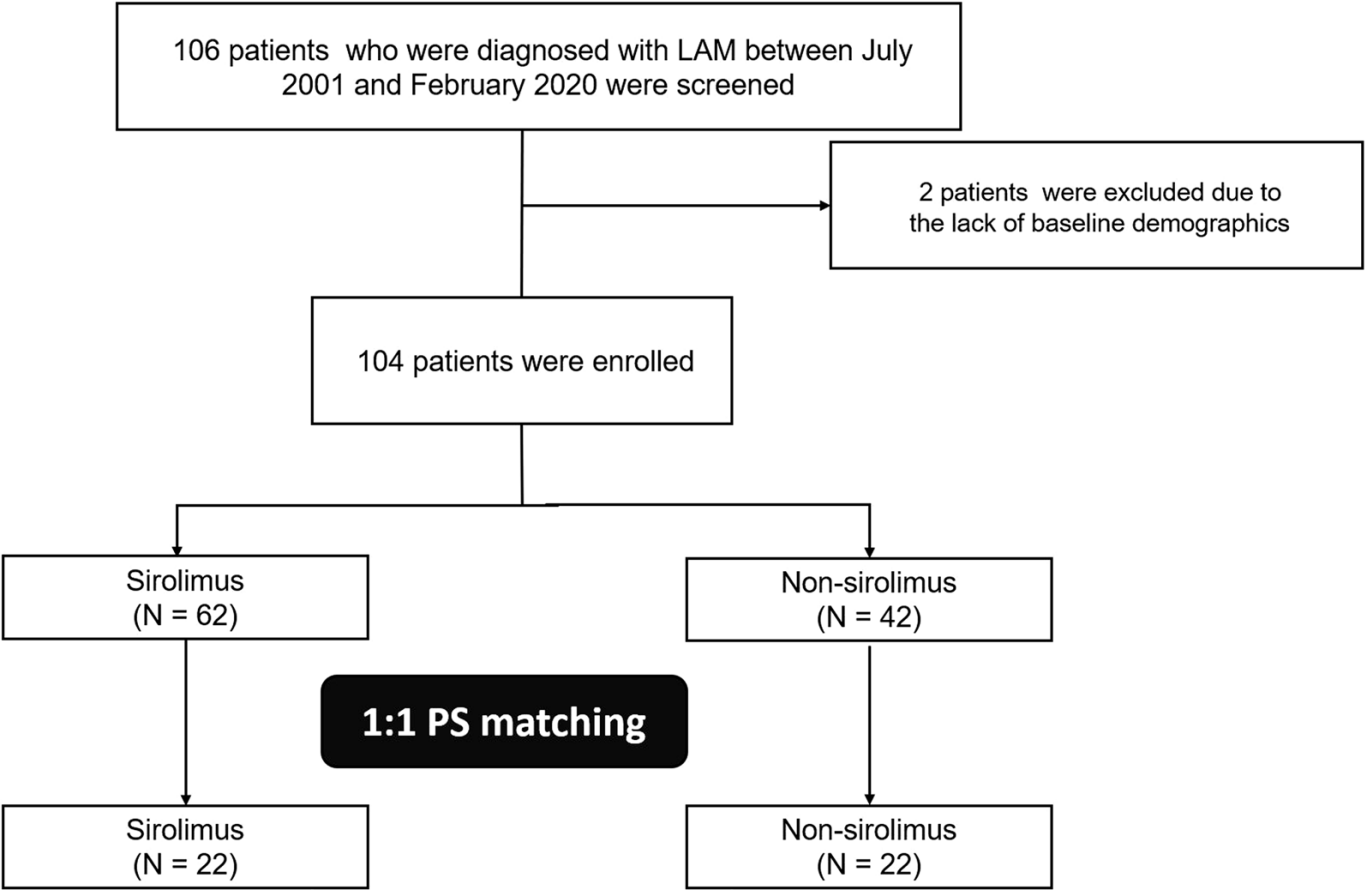
Flow chart of the patient selection. *LAM* lymphangioleiomyomatosis, *PS* propensity score

Of a total of 106 patients who were diagnosed with LAM between July 2001 and February 2020 at Asan Medical Center, Seoul, Republic of Korea, 104 patients whose baseline data were available, were included in this study (Fig. 1). All patients met the diagnostic criteria of definite LAM according to An Official American Thoracic Society/Japanese Respiratory Society Clinical Practice Guideline [1]. Of all the included cases, 60.6% were biopsy-proven through surgical lung biopsy (48.1%), transbronchial lung biopsy (9.6%), or biopsy of other sites including the retroperitoneum and lymph nodes (2.9%). The study protocol was approved by the Institutional Review Board of Asan Medical Center (2016-0480). Informed consent was not deemed necessary owing to the retrospective nature of the study.

### Clinical data

The clinical and survival data of all patients were retrospectively collected from medical records, telephone interviews, and records from the National Health Insurance of South Korea. Spirometry and measurement of DLCO and TLC were performed according to the recommendations of the ATS and European Respiratory Society [9–11]; and the results are expressed as a percentage of the normal predicted values. The 6-min walk test (6MWT) was performed based on the ATS guidelines [12].

Records of follow-up visits which took place every 3–6 months, and hospitalization were reviewed to identify the development of complications such as disease progression (DP) and extrapulmonary involvement. DP was defined as a 10% absolute decline in FEV_1_ from baseline values.

### Statistical analysis

Continuous variables are expressed as mean ± standard deviation, while categorical variables are expressed as percentages. The student’s t-test or the Mann–Whitney U test was used to analyze continuous data, whereas the Pearson’s chi-square test or Fisher’s exact test was used to analyze categorical data. Death was defined as the primary outcome, and lung transplantation was considered an equivalent outcome to death. The Cox regression model was used to identify the prognostic factors for survival and variables with *P* < 0.1 in the unadjusted analysis were incorporated in the multivariable model using backward elimination. Survival was evaluated using the Kaplan–Meier survival analysis and the log-rank test. The availability of serial lung function data (≥ 3 measurements) in 92 patients enabled the calculation of the annual rate (slope) of decline in lung function using a linear regression model.

All patients were divided into two (sirolimus and non-sirolimus) groups according to whether or not they received sirolimus therapy, to evaluate its effect on the clinical outcome of patients with LAM. The index date was set as the date of the first prescription of sirolimus in the sirolimus group and the date of LAM diagnosis in the non-sirolimus group. Patients were observed from the index date until the occurrence of the study outcome or until the end of the observation period (April 30, 2020), whichever took place earlier. Propensity score matching (1:1) to adjust for the differences in baseline characteristics between the sirolomus and non-sirolimus groups, yielded 22 matched pairs (Fig. 1). The matched variables consisted of age, history of smoking, FEV_1_, and DLCO; propensity scores were calculated using the multiple logistic regression analysis to estimate the probability of receiving sirolimus therapy in each patient. All statistical analyses were performed using the SPSS software version 23.0 (IBM Inc., Chicago, IL, USA). All *P*-values were two-tailed, and *P*-values < 0.05 were considered statistically significant.

## Results

### Baseline characteristics at diagnosis

**Fig. 2:**
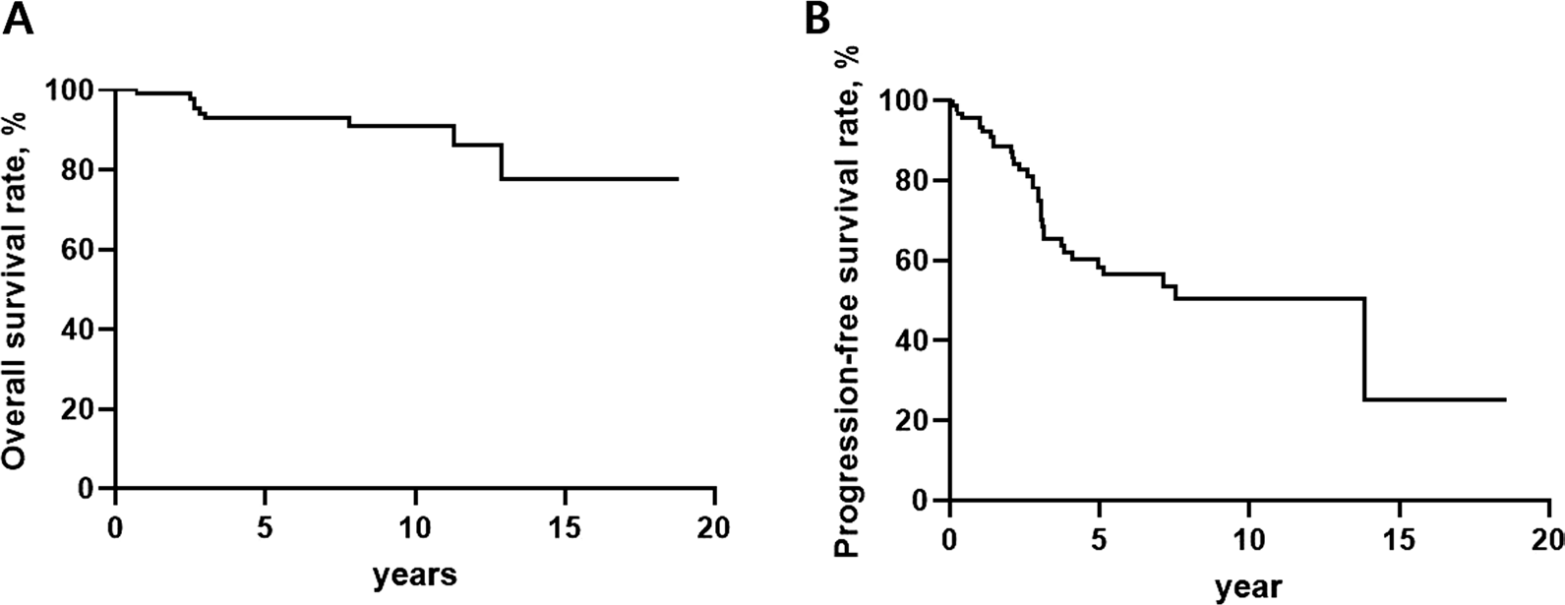
Kaplan–Meier curves for overall survival and disease progression in patients with LAM. **A** Survival curve for overall survival. **B** Survival curve for disease progression. *LAM* lymphangioleiomyomatosis

Among all patients, the mean age was 40.3 years, 9.6% were ever-smokers, and 12.5% were diagnosed with tuberous sclerosis complex (TSC) (Table 1). During follow-up (median: 7.1 years, interquartile range: 2.8–9.9 years), seven (6.7%) patients died, and two patients (1.9%) underwent lung transplantation. The 5-year and 10-year survival rates were 93.0% and 90.9%, respectively (Fig. 2A). The non-survivors had lower lung function as manifested by FEV_1_, DLCO and FEV_1_/FVC, higher values of residual volume (RV), and poorer exercise capacity as shown by the distance and the lowest oxygen saturation (SpO_2_) during the 6MWT, than the survivors (Table 1). However, there were no significant differences in the incidence of pneumothorax and extrapulmonary manifestations. Sirolimus was administered to 59.6% of patients, and no significant difference in the treatment received was observed between the non-survivors and survivors.

**Table 1:** Comparison of baseline characteristics and treatment between the non-survivors and survivors among patients with LAM

Characteristic	Total	Non-survivors	Survivors	*P*-value
Number of patients	104	9	95	
Age, years	40.3 ± 10.7	45.8 ± 13.0	39.8 ± 9.9	0.178
Female	104 (100)	9 (100)	95 (100)	> 0.999
Ever-smoker	10 (9.6)	1 (11.1)	9 (9.5)	> 0.999
TSC	13 (12.5)	1 (11.1)	12 (12.6)	> 0.999
Pneumothorax	44 (42.3)	4 (44.4)	40 (42.1)	0.944
Chylothorax	2 (1.9)	0 (0.0)	2 (2.1)	> 0.999
Extrapulmonary manifestations	48 (46.2)	4 (44.4)	44 (46.3)	> 0.999
Angiomyolipoma	32 (30.5)	3 (33.3)	29 (30.5)	> 0.999
Lymphangioleiomyoma	18 (17.3)	1 (11.1)	17 (17.9)	> 0.999
Lung function, % predicted				
FEV_1_	77.3 ± 22.2	55.4 ± 24.7	79.3 ± 19.6	0.005
FVC	88.5 ± 16.8	88.1 ± 21.3	88.6 ± 13.8	0.939
DLCO	63.5 ± 249	40.1 ± 19.7	65.7 ± 23.4	0.003
TLC	97.8 ± 16.5	104.9 ± 18.0	97.3 ± 12.9	0.102
FEV_1_/FVC	87.3 ± 19.1	64.8 ± 25.2	89.4 ± 17.1	0.003
RV	102.3 ± 35.5	123.9 ± 24.2	99.5 ± 28.8	0.010
FEF_25–75%_	67.3 ± 29.9	29.6 ± 21.8	71.0 ± 39.5	0.448
6MWT				
Distance, m	467.5 ± 114.6	320.5 ± 168.9	481.0 ± 86.5	0.004
Lowest SpO_2_, %	94.4 ± 5.8	87.4 ± 12.2	95.0 ± 4.4	0.048
Treatment				
Medroxyprogesterone	31 (29.8)	4 (44.4)	27 (28.4)	0.446
LHRH	2 (1.9)	0 (0.0)	2 (2.1)	> 0.999
Bilateral oophorectomy	5 (4.8)	0 (0.0)	5 (5.3)	> 0.999
Sirolimus therapy	62 (59.6)	4 (44.4)	58 (61.1)	0.480
Dose per day, mg	1.6 ± 0.6	1.4 ± 0.5	1.6 ± 0.6	0.490
TDM, ng/mL	5.9 ± 3.0	4.3 ± 1.8	6.0 ± 3.1	0.361
Duration, years	4.8 (1.5–6.2)	1.5 (0.3–3.4)	5.0 (1–6.7)	0.054

Data are expressed as the mean ± standard deviation or as a number (%), unless otherwise indicated

*DLCO* diffusing capacity of the lung for carbon monoxide, *FEV*_*1*_ forced expiratory volume in one second, *FVC* forced vital capacity, *RV* residual volume, *FEF*_*25–75%*_ forced expiratory flow between 25 and 75% of FVC, *LAM* lymphangioleiomyomatosis, *SpO*_*2*_ oxygen saturation, *TLC* total lung capacity, *TSC* tuberous sclerosis complex, *6MWT* 6-min walk test, *LHRH* luteinizing hormone-releasing hormone, *TDM* therapeutic drug monitoring

### Clinical course

During follow-up, 33 patients (35.9% of the patients with serial lung function data [≥ 3 measurements; n = 92]) experienced DP (Table 2). The 5-year and 10-year progression-free survival rates were 61.2% and 56.2%, respectively (Fig. 2B). Among the complications detected after the diagnosis of LAM, pneumothorax and angiomyolipoma (AML) were the most common (in each 2.9%), followed by chylothorax (1.9%) (Table 2). Analysis of the serial lung function data (n = 92), revealed a mean annual decline rate in FEV_1_, FVC, and DLCO of − 0.7 ± 4.2% predicted/year, 0.3 ± 3.2% predicted/year, and 1.5 ± 4.0% predicted/year, respectively (Table 2).

**Table 2:** Comparison of clinical course between the non-survivors and survivors among patients with LAM

Characteristics	Total	Non-survivors	Survivors	*P*-value
Number of patients	104	9	95	
Pneumothorax	3 (2.9)	0 (0.0)	3 (3.2)	> 0.999
Chylothorax	2 (1.9)	0 (0.0)	2 (2.1)	> 0.999
Extrapulmonary manifestations	3 (2.9)	0 (0.0)	3 (3.2)	> 0.999
Angiomyolipoma	3 (2.9)	0 (0.0)	3 (3.2)	> 0.999
Lymphangioleiomyoma	0 (0.0)	0 (0.0)	0 (0.0)	> 0.999
Disease progression	33/92 (35.9)	4/7 (57.1)	29/85 (34.1)	0.245
Lung function decline	N = 92	N = 7	N = 85	
FEV_1_, % predicted/year	− 0.7 ± 4.2	− 2.0 ± 5.4	− 0.7 ± 4.1	0.448
FVC, % predicted/year	0.3 ± 3.2	− 2.2 ± 4.3	0.8 ± 3.1	0.147
DLCO, % predicted/year	1.5 ± 4.0	− 1.1 ± 3.6	− 1.5 ± 4.1	0.929
RV, % predicted/year	− 1.0 ± 9.1	2.7 ± 4.9	− 1.3 ± 9.3	0.221
FEF_25–75_, % predicted/year	− 2.4 ± 7.4	1.6 ± 8.8	− 2.8 ± 7.2	0.185
Follow-up period, year	7.1 (2.8–9.9)	2.7 (2.5–9.6)	7.2 (3.0–9.9)	0.236

Data are expressed as the mean ± standard deviation, a number (%) or the median (interquartile range), unless otherwise indicated

*DLCO* diffusing capacity of the lung for carbon monoxide, *FEV*_*1*_ forced expiratory volume in one second, *FVC* forced vital capacity, *RV* residual volume, *FEF*_*25–75%*_ forced expiratory flow between 25 and 75% of FVC, *LAM* lymphangioleiomyomatosis

No significant differences in the development of complications (pneumothorax, chylothorax, and extrapulmonary manifestations) after the diagnosis of LAM, DP, and lung function decline rate were noted between the non-survivors and survivors (Table 2).

### Risk factors for mortality

The unadjusted Cox analysis demonstrated that lower lung function (FEV_1_, FVC, DLCO, TLC, FEV_1_/FVC, and FEF_25–75%_ [forced expiratory flow between 25 and 75% of FVC]) and poorer exercise capacity (distance and the lowest SpO_2_ during the 6MWT) were associated with mortality in patients with LAM (Table 3). Due to the close correlation between FEV_1_ and DLCO (r = 0.705), we developed two multivariable models with each including either FEV_1_ or DLCO. The multivariable model including age, FEV_1_, 6-min walk distance (6MWD), and lowest SpO_2_ during the 6MWT showed that older age (hazard ratio [HR]: 1.136, 95% confidence interval [CI]: 1.016–1.269, *P* = 0.025), lower FEV_1_ (HR: 0.956, 95% CI: 0.919–0.995, *P* = 0.026), and shorter 6MWD (HR: 0.993, 95% CI: 0.987–0.999, *P* = 0.020) were independent prognostic factors for mortality in patients with LAM. The other multivariable model including age, DLCO, 6WMD, and lowest SpO_2_ during the 6MWT showed that older age (HR: 1.167, 95% CI: 1.036–1.314, *P* = 0.011), and lower DLCO (HR: 0.914, 95% CI: 0.861–0.969, *P* = 0.003) were also risk factors for mortality.

**Table 3:** Prognostic factors for mortality in patients with LAM assessed using the Cox proportional analysis

Characteristics	Unadjusted		Multivariable model-1		Multivariable model-2	
	HR (95% CI)	*P*-value	HR (95% CI)	*P*-value	HR (95% CI)	*P*-value
Age	1.046 (0.993–1.102)	0.090	1.136 (1.016–1.269)	0.025	1.167 (1.036–1.314)	0.011
Ever-smoker	1.388 (0.172–11.178)	0.758				
TSC	0.949 (0.117–7.689)	0.961				
Pneumothorax	0.920 (0.246–3.439)	0.902				
Angiomyolipoma	1.149 (0.285–4.627)	0.845				
FEV_1_	0.956 (0.927–0.984)	0.003	0.956 (0.919–0.995)	0.026		
FVC	1.000 (0.954–1.048)	0.996				
DLCO	0.946 (0.914–0.979)	0.001			0.914 (0.861–0.969)	0.003
FEV_1_/FVC ratio	0.947 (0.919–0.976)	< 0.001				
RV	1.007 (0.996–1.019)	0.214				
FEF_25–75_	0.946 (0.936–0.993)	0.014				
6MWD	0.992 (0.987–0.996)	< 0.001	0.993 (0.987–0.999)	0.020	0.997 (0.992–1.002)	0.247
6MWT, the lowest SpO_2_, %	0.921 (0.868–0.977)	0.006	1.007 (0.908–1.117)	0.890	0.994 (0.902–1.095)	0.905
Sirolimus therapy	0.584 (0.155–2.195)	0.426				

FEV_1_ and DLCO were separately included in the multivariable models due to the high correlation between them (r = 0.705). FEV_1_/FVC (r = 0.803), and FEF_25-75_ (r = 0.767) were not included in the multivariable models due to their correlation with FEV_1_

*CI* confidence interval, *DLCO* diffusing capacity of the lung for carbon monoxide, *FEV*_*1*_ forced expiratory volume in 1 s, *FVC* forced vital capacity, *RV* residual volume, *FEF*_*25–75%*_ forced expiratory flow between 25 and 75% of FVC, *HR* hazard ratio, *LAM* lymphangioleiomyomatosis, *SpO*_*2*_ oxygen saturation, *TLC* total lung capacity, *TSC* tuberous sclerosis complex, *6MWD* 6-min walk distance, *6MWT* 6-min walk test

### Effects of sirolimus therapy

In the unmatched analysis, patients treated with sirolimus were younger, had a higher incidence of lymphangioleiomyoma, lower lung function (FEV_1_, DLCO, FEV_1_/FVC, and FEF_25-75%_), and poorer exercise capacity (distance and lowest SpO_2_ during the 6MWT) than those who did not receive sirolimus therapy (Additional file 1: Table S1). Propensity score matching that was performed to adjust for these differences generated 22 matched pairs (Table 4). The baseline characteristics of the patients in the matched cohort and those in the unmatched one are displayed in Additional file 1: Table S2. The unmatched patients had a higher incidence of lymphangioleiomyoma and a shorter distance during the 6MWT. Additionally, the lowest SpO_2_ during the 6MWT was inferior in the unmatched patients compared with the matched ones.

**Table 4:** Comparison of baseline characteristics between the sirolimus and the non-sirolimus groups among patients with LAM

Characteristic	Sirolimus	Non-sirolimus	*P*-value
Number of patients	22	22	
Age, years	42.4 ± 3.2	40.8 ± 9.2	0.723
Female sex	22 (100)	22 (100)	> 0.999
Ever-smoker	1 (4.5)	3 (13.6)	0.607
TSC	2 (9.1)	3 (13.6)	> 0.999
Pneumothorax	12 (54.5)	9 (40.9)	0.365
Chylothorax	0 (0.0)	2 (9.1)	0.488
Extrapulmonary manifestations	7 (31.8)	10 (45.5)	0.537
Angiomyolipoma	7 (31.8)	7 (31.8)	> 0.999
Lymphangioleiomyoma	1 (4.5)	2 (9.1)	> 0.999
Lung function, % predicted			
FEV_1_	75.0 ± 18.0	78.1 ± 20.1	0.655
FVC	85.9 ± 12.9	87.1 ± 13.6	0.796
DLCO	60.9 ± 18.0	62.2 ± 15.5	0.655
TLC	97.5 ± 11.9	96.0 ± 12.5	0.879
FEV_1_/FVC	87.5 ± 16.5	90.3 ± 20.0	0.348
RV	98.6 ± 23.3	101.4 ± 22.3	0.702
FEF_25–75%_	75.4 ± 42.8	76.7 ± 47.2	0.930
6MWT			
Distance, m	513.4 ± 79.6	508.0 ± 54.2	0.496
Lowest SpO_2_, %	93.9 ± 4.0	95.7 ± 5.0	0.047

Data are expressed as the mean ± standard deviation or as a number (%), unless otherwise indicated. The index date was set as the date of the first prescription of sirolimus in the sirolimus group and the date of LAM diagnosis in the non-sirolimus group

*DLCO* diffusing capacity of the lung for carbon monoxide, *FEV*_*1*_ forced expiratory volume in one second, *FVC* forced vital capacity, *RV* residual volume, *FEF*_*25–75%*_ forced expiratory flow between 25 and 75% of FVC, *LAM* lymphangioleiomyomatosis, *TSC* tuberous sclerosis complex, *TLC* total lung capacity, *SpO*_*2*_ oxygen saturation, *6MWT* 6-min walk test

Among the matched patients, the mean age was 40.7 years and TSC was diagnosed in 11.4% patients. The median follow-up duration was 7.1 years (8.5 years in the sirolimus group versus 6.1 years in the non-sirolimus group, *P* = 0.213). Additionally, the median time from diagnosis to the start of sirolimus therapy was 1.8 (interquartile range: 0.2–3.8) years, and the median duration of sirolimus therapy was 4.5 (interquartile range: 1.5–6.0) years. Although the sirolimus group showed lower the minimum SpO_2_ during the 6MWT than the non-sirolimus group (Table 4), there were no differences in survival, DP, and lung function decline rate between the two groups (Table 5). Moreover, in the Kaplan–Meier survival analysis, there was a tendency of better survival in the sirolimus group than that in the no-sirolimus group (P = 0.073) (Additional file 1: Fig. S1).

**Table 5:** Comparison of clinical course between the sirolimus and non-sirolimus groups among patients with LAM

Characteristics	Sirolimus	Non-sirolimus	*P*-value
Number of patients	22	22	
Death or lung transplantation	0 (0.0)	3 (13.6)	0.233
Disease progression	4/21 (19.0)	6/22 (27.3)	0.721
Lung function decline, % predicted/year	N = 17	N = 19	
FEV_1_, % predicted/year	0.5 ± 3.0	− 0.3 ± 4.7	0.452
FVC, % predicted/year	0.7 ± 3.1	0.0 ± 4.1	> 0.999
DLCO, % predicted/year	− 1.2 ± 3.9	0.6 ± 5.5	0.271
RV, % predicted/year	0.1 ± 5.8	− 3.4 ± 15.3	0.485
FEF_25-75%_, % predicted/year	− 1.3 ± 4.1	− 5.7 ± 13.9	0.808
Median follow-up period, year	8.5 (3.8–11.5)	6.1 (2.4–9.5)	0.213

Data are expressed as the mean ± standard deviation, a number (%) or the median (interquartile range), unless otherwise indicated

*DLCO* diffusing capacity of the lung for carbon monoxide, *FEV*_*1*_ forced expiratory volume in one second, *FVC* forced vital capacity, *RV* residual volume, *FEF*_*25–75%*_ forced expiratory flow between 25 and 75% of FVC, *IQR* interquartile range, *LAM* lymphangioleiomyomatosis

## Discussion

This study reported the long-term clinical course and prognostic factors of patients with LAM. The 10-year survival and progression-free survival rates were 90.9% and 56.2%, respectively. Older age, lower FEV_1_ and DLCO, and shorter 6MWD were independent prognostic factors for overall survival in patients with LAM.

In our study, the prognosis of patients with LAM was better than that reported previously [3, 4, 13, 14]. The studies published in the 2000s (n = 57–105) demonstrated a mortality rate of 10–20% after symptom onset during median follow-up period of 4.5–12.6 years [4, 14]. Oprescu et al. reported a 10-year transplantation-free survival of 86% in 410 patients with LAM who were followed up over a median time period of 10.4 years until 2007 [3]. Gupta et al., who observed 217 patients with LAM until 2014, also demonstrated 5-year and 10-year transplantation-free survival rates of 94% and 85%, respectively [5]. Having followed up patients with LAM until April 2020, we found a higher 10-year transplantation-free survival rate of 90.9%. The better survival of patients in our study might be attributed to improved management of patients with LAM, and more widespread access to chest computed tomography imaging for screening purposes in South Korea, which could have led to earlier diagnosis of LAM.

Our study revealed that older age and lower lung function (FEV_1_ or DLCO) were independent prognostic factors for mortality in patients with LAM. The prognostic factors in patients with LAM have been previously reported [3, 5–7]. The age-adjusted multivariable Cox analysis performed by Gupta et al. revealed that lower FEV_1_ (HR: 0.97, 95% CI: 0.96–0.99, *P* = 0.008) and DLCO (HR: 0.97, 95% CI: 0.95–0.99, *P* = 0.001) were independent risk factors for transplantation-free survival [5]. The multivariable Cox analysis conducted by Oprescu et al., also showed that late symptom onset after diagnosis (HR: 0.80, 95% CI: 0.64–0.99, *P* = 0.043), and the presence of AML (HR: 0.49, 95% CI: 0.30–0.79, *P* = 0.004) were associated with a lower risk of death or transplantation, while home oxygen use (HR: 3.13, 95% CI: 1.90–5.18, *P* < 0.001) was associated with poor prognosis [3]. These findings support our results which have shown an association between higher disease severity and poor prognosis. On the other hand, Kitaichi et al. revealed that a lower FEV_1_/FVC ratio and a higher TLC were associated with poor prognosis in a study performed on 46 patients with LAM [7]. These findings are also consistent with our results; lower FEV_1_/FVC and increased TLC were associated with a higher risk of mortality in the unadjusted analysis. The lower FEV_1_/FVC ratio reflects airflow limitation [15], while an increase in TLC indicates hyperinflation of the lungs due to air trapping [16] in obstructive lung disease.

After propensity score matching for age, smoking status, and lung function, no significant differences in clinical outcome and lung function decline rate were found between the sirolimus and non-sirolimus groups. However, these results actually suggest the presence of clinical benefits with sirolimus therapy in patients with LAM, since the sirolimus group included patients with more advanced disease (lower the minimum SpO_2_ during 6MWT). The efficacy of sirolimus in the stabilization of lung function has been demonstrated by real-world studies [17–20]. In a study performed on 98 Chinese patients with LAM, Zhan et al., showed an improvement in FEV_1_ decline (n = 18; − 31.1 ± 30.8 [pre-sirolimus] versus 16.1 ± 36.0 mL/month [post-sirolimus], *P* = 0.002), 6MWD (n = 46; 358.8 ± 114.4 versus 415.6 ± 118.6 m, *P* = 0.004), and arterial blood oxygen tension (n = 17; − 0.55 ± 0.60 versus 0.30 ± 1.19 mmHg/month, *P* = 0.018) [17]. Bee et al. also demonstrated an improvement in FEV_1_ after sirolimus therapy (− 150 versus 35 mL/year, n = 21, *P* < 0.001) in a prospective national cohort study conducted on 47 patients with LAM in the UK [18]. Through the Multicenter International Lymphangioleiomyomatosis Efficacy of Sirolimus trial, Gupta et al. demonstrated that sirolimus therapy stabilized FEV_1_ decline regardless of clinical features including menopausal status, baseline FEV_1_, and co-existing TSC [20]. Despite the lack of statistical significance, the rate of FEV_1_ decline in the sirolimus group was numerically lower (0.5 versus − 0.3% predicted/year) than that in the non-sirolimus group in our study.

Our study had a few limitations. First, the generalizability of our results could be limited by the single-centered and retrospective nature of the study. Nonetheless, the baseline characteristics of our patients were similar to those of previous studies [21]. Second, the small number of patients analyzed (the non-survivors and the matched patients) could account for the lack of statistical significance. However, our analyses suggested prognostic factors consistent with previous studies [3, 5–7]. Third, the patients were enrolled over a long period of time. Standards of care for patients with LAM might have evolved over time. However, despite these limitations, the strengths of our study include its long-term follow-up period and the determination of prognostic factors based on various clinical variables.

## Conclusion

During long-term follow-up of about 7 years, a tenth, and a third of patients with LAM experienced death or DP. Our results suggest that older age, lower lung function, and poorer exercise capacity mean poor prognosis in patients with LAM.

## Supplementary Information

**Additional file 1: Table S1.** Comparison of baseline characteristics between the sirolimus and non-sirolimus groups among patients with LAM. **Table S2.** Comparison of baseline characteristics between propensity score-matched and unmatched groups among patients with LAM. **Figure S1.** Comparison of survival curves between the sirolimus and non-sirolimus groups among the matched patients with LAM.

## Data Availability

Any data generated and/or analysed during the current study are available from the corresponding author on reasonable request.

## Abbreviations

DLCO: Diffusing capacity of the lung for carbon monoxide
DP: Disease progression
FEV_1_: Forced expiratory volume in one second
FVC: Forced vital capacity
LAM: Lymphangioleiomyomatosis
SpO_2_: Oxygen saturation
TLC: Total lung capacity
TSC: Tuberous sclerosis complex
6MWD: 6-Minute walk distance
6MWT: 6-Minute walk test
