# Supplementary material for: Long-term clinical course and outcomes in patients with lymphangioleiomyomatosis
Source: Respir Res. 2022 Jun 18;23:158. doi: 10.1186/s12931-022-02079-6 (PMC9206248; doi:10.1186/s12931-022-02079-6)
Supplement: Supplementary file 1 — Additional file 1: Table S1. Comparison of baseline characteristics between the sirolimus and non-sirolimus groups among patients with LAM. Table S2. Comparison of baseline characteristics between propensity score-matched and unmatched groups among patients with LAM. Figure S1. Comparison of survival curves between the sirolimus and non-sirolimus groups among the matched patients with LAM. [file 12931_2022_2079_MOESM1_ESM.docx]

**Long-term clinical course and outcomes in patients with lymphangioleiomyomatosis**

Hee-Young Yoon^1^, Ho Jeong Kim^2^, Jin Woo Song^2*^

^1^Division of Internal Medicine, Seoul North Municipal Hospital

^2^Department of Pulmonary and Critical Care Medicine, Asan Medical Center, University of Ulsan College of Medicine, Seoul, Republic of Korea

***Corresponding author**

**Table S1.** Comparison of baseline characteristics between the sirolimus and non-sirolimus groups among patients with LAM.

| Characteristic | Sirolimus | Non-sirolimus | *P*-value |
| --- | --- | --- | --- |
| Number of patients | 62 | 42 |  |
| Age, years | 38.6 ± 8.9 | 44.6 ± 10.5 | 0.002 |
| Female sex | 62 (100) | 42 (100) | > 0.999 |
| Ever-smoker | 8 (12.9) | 2 (4.8) | 0.197 |
| TSC | 10 (16.1) | 3 (7.1) | 0.174 |
| Pneumothorax | 27 (43.5) | 18 (42.9) | 1.000 |
| Chylothorax | 4 (6.5) | 0 (0.0) | 0.146 |
| Extrapulmonary manifestations | 37 (59.7) | 12 (28.6) | 0.003 |
| Angiomyolipoma | 21 (33.9) | 12 (28.6) | 0.669 |
| Lymphangioleiomyoma | 17 (27.4) | 1 (2.4) | 0.001 |
| Lung function, % predicted |  |  |  |
| FEV_1_ | 69.5 ± 20.7 | 83.4 ± 17.6 | 0.001 |
| FVC | 87.0 ± 14.3 | 90.0 ± 13.6 | 0.274 |
| DLCO | 51.1 ± 21.2 | 74.0 ± 19.4 | < 0.001 |
| TLC | 97.7 ± 14.2 | 98.0 ± 12.4 | 0.905 |
| FEV_1_/FVC | 79.8 ± 20.0 | 93.2 ± 16.6 | 0.001 |
| RV | 105.3 ± 41.0 | 97.6 ± 24.5 | 0.295 |
| FEF_25-75%_ | 60.5 ± 38.3 | 77.6 ± 40.7 | 0.035 |
| 6MWT |  |  |  |
| Distance, m | 445.6 ± 123.8 | 504.2 ± 56.1 | 0.029 |
| Lowest SpO_2_, % | 91.6 ± 6.4 | 96.2 ± 4.1 | < 0.001 |

Data are expressed as the mean ± standard deviation or as a number (%), unless otherwise indicated. The index date was set as the date of the first prescription of sirolimus in the sirolimus group and the date of LAM diagnosis in the non-sirolimus group.

DLCO, diffusing capacity of the lung for carbon monoxide; FEV_1_, forced expiratory volume in one second; FVC, forced vital capacity; LAM, lymphangioleiomyomatosis; SpO_2_, oxygen saturation; TLC, total lung capacity; RV, residual volume; FEF_25-75%_, forced expiratory flow between 25% and 75% of FVC; TSC, tuberous sclerosis complex; 6MWT, 6-minute walk test.

**Table S2.** Comparison of baseline characteristics between propensity score-matched and unmatched groups among patients with LAM.

| Characteristics | Matched | Unmatched | *P*-value |
| --- | --- | --- | --- |
| Number of patients | 44 | 60 |  |
| Age, years | 40.7 ± 8.7 | 40.0 ± 11.0 | 0.738 |
| Female sex | 44 (100.0) | 60 (100.0) | > 0.999 |
| Ever-smoker | 4 (9.1) | 6 (10.0) | > 0.999 |
| TSC | 5 (11.4) | 8 (13.3) | 0.764 |
| Pneumothorax | 20 (45.5) | 24 (40.0) | 0.578 |
| Chylothorax | 0 (0.0) | 2 (3.3) | 0.507 |
| Extrapulmonary manifestations | 16 (36.4) | 32 (53.3) | 0.086 |
| Angiomyolipoma | 13 (29.5) | 19 (31.7) | 0.817 |
| Lymphangioleiomyoma | 3 (6.8) | 15 (25.0) | 0.015 |
| Lung function, % predicted |  |  |  |
| FEV_1_ | 79.4 ± 19.4 | 75.8 ± 22.1 | 0.395 |
| FVC | 87.9 ± 12.9 | 89.0 ± 15.6 | 0.712 |
| DLCO | 64.3 ± 17.7 | 62.9 ± 29.2 | 0.780 |
| TLC | 96.8 ± 12.1 | 98.6 ± 14.5 | 0.493 |
| FEV_1_/FVC | 90.6 ± 18.1 | 84.8 ± 19.6 | 0.124 |
| RV | 99.9 ± 22.6 | 104.1 ± 43.0 | 0.572 |
| FEF_25-75%_ | 76.0 ± 44.5 | 61.0 ± 35.4 | 0.062 |
| 6MWT |  |  |  |
| Distance, m | 495.6 ± 75.6 | 445.1 ± 119.2 | 0.019 |
| Lowest SpO_2_, % | 96.0 ± 4.2 | 93.0 ± 6.7 | 0.011 |
| Sirolimus | 22 (50.0) | 40 (66.7) | 0.107 |
| Median follow-up period, year (IQR) | 7.1 (2.6–10.3) | 7.1 (2.8–9.7) | 0.655 |

Data are expressed as the mean ± standard deviation or as a number (%), unless otherwise indicated.

DLCO, diffusing capacity of the lung for carbon monoxide; FEV_1_, forced expiratory volume in one second; FVC, forced vital capacity; RV, residual volume; FEF_25-75%_, forced expiratory flow between 25% and 75% of FVC; LAM, lymphangioleiomyomatosis; SpO_2_, oxygen saturation; TLC, total lung capacity; TSC, tuberous sclerosis complex; 6MWT, 6-minute walk test.

**Figure legends**

**Figure S1.** Comparison of survival curves between the sirolimus and non-sirolimus groups among the matched patients with LAM


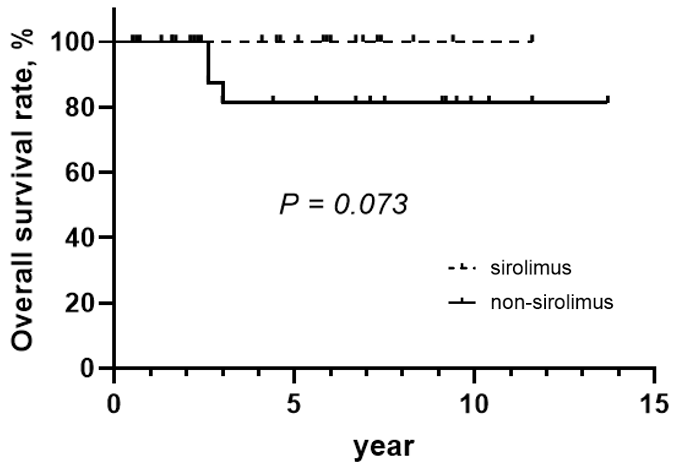


LAM, lymphangioleiomyomatosis
